# The elephant in the room: reproducibility in toxicology

**DOI:** 10.1186/s12989-014-0042-8

**Published:** 2014-08-22

**Authors:** Craig A Poland, Mark R Miller, Rodger Duffin, Flemming Cassee

**Affiliations:** 1 Institute of Occupational Medicine, Research Avenue North Riccarton, Edinburgh EH14 4AP, UK; 2 University/BHF Centre for Cardiovascular Science, University of Edinburgh, 47 Little France Crescent, Edinburgh, EH16 4TJ, Scotland, UK; 3 MRC Centre for Inflammation Research, University of Edinburgh, Edinburgh, Scotland, UK; 4 Centre for Sustainability & Environmental Health, National Institute for Public Health and the Environment (RIVM), Bilthoven, The Netherlands; 5 Institute for Risk Assessment Sciences, Utrecht University, Utrecht, The Netherlands

## Abstract

No abstract.

## 

**The issue of reproducibility of results in toxicology has long been a concern and, perhaps, at the back of many of our minds but not necessarily at the forefront of our thinking – ‘the elephant in the room’. Are the results we have published literally*our*results or are they reproducible and truly part of a credible theory?** In the excellent editorial by Gary Miller [1], Editor-in-Chief of the journal *Toxicological Sciences*, he discusses the issue of reproducibility in scientific manuscripts; an important topic and one that has received increasing attention of late from researchers, journals and funding bodies.

## The problem?

The editorial on the topic of reproducibility, or lack of, in toxicology follows on from concerns raised in the journal *Nature*[2],[3], including a collation of publications and comments on the journals website (http://www.nature.com/nature/focus/reproducibility/). In addition in January this year, the Director and Deputy Director of National Institute of Health (NIH) outlined what the NIH plans to do to tackle the problem [4]. Of greatest concern, is the contribution poor reproducibility can have on the attrition rates of potential therapies as they move from preclinical testing (*in situ*, *in vitro* and *in vivo*) to rigorous clinical trials. Clinical trials require specific levels of stringency (e.g. power estimates, blinding, randomisation, etc. [4]) that are not always observed within basic research. Whilst the issue of attrition, or the failure of novel therapies to reach their full potential, pertains to a sub-set of scientific endeavour, the issue of irreproducibility impacts on *all* areas of science. This can range from public (mis)trust of science to the unintentional squandering of increasingly limited time and funds pursuing blind avenues. The implications of this should not be underestimated. Research agendas, whilst ultimately driven by the researcher, are heavily influenced by funding bodies, public opinion and political motivations. All will play a role in the distribution of a limited pool of research funds, and rightly expect progress in return for financial support. Yet a lack of reproducibility can destabilise research and undermine the confidence of stakeholders. An additional area of concern relating to reproducibility is when findings are translated into the ‘real world’ where acceptance and use of premature conclusions resulting from uncertain data [5] can have profound implications. This may be in the form of turning the tide of trust towards or against new technologies (if unfounded) or where regulatory decisions are made based on shaky foundations. As stated by Gio Batta Gori, Editor-in-Chief of the Journal *Regulatory Toxicology and Pharmacolgy*[5], this is not just a scientific issue but also an ethical one; how uncertain results are presented to, interpreted and consumed by non-academic organisations.

## The cause?

As with the Editorials cited above [1]–[4], it is important to clearly state that this is not a question of scientific misconduct, but instead often one of an increasing number of simple mistakes in the research design. These may occur everywhere from poorly designed protocols, incorrect controls, underpowered studies, improper data analysis/reporting (such as the use of error bars or confidence intervals [6]), the differentiation between technical replicates and independent experiments [7],[8], as well as inaccurate/incomplete reporting of methods [7]. This latter issue has been suggested to be especially crucial, as even minor modifications of conventional *in vitro* toxicity assays (e.g. due to sample handling/ preparation issues) can have an important impact on study reproducibility [9].

These and many more factors harm the reproducibility of studies. However, the articles on reproducibility cited above do not advocate a ‘thou shalt’ approach, which is something we at *Particle and Fibre Toxicology* agree with. Instead these articles wish to offer reasons as to why some basic standards may have slipped, and suggest ways to improve reproducibility that could be useful to us all. The articles point out that it is not just the individual researcher or senior investigators that are at fault, but also that motivations in sciences are too heavily influenced by a ‘rewards system’ whereby large scale, high volume, low cost studies are incentivised, and continued publication in high-profile journals is, more-than-ever, a necessity – “publish or perish”. These factors can improve scientific progress, pushing up standards and output/ pushing down costs, however there is a clear risk that the intensity and speed of this competitive approach could come at the cost of scientific rigour.

One commenter on the 2012 *Nature* editorial [2] offered that “one could say there is no way to have real science and money hand in hand especially when one is required to generate money with the respective science”. Many will recognise the truth in such a statement, although others would argue that this is the environment we inhabit and one that is increasingly unlikely to change. Today’s research environment is increasingly based on short-term contracts whereby acquisition of research grants and publications are the main two drivers (certainly from an Academic perspective) that offer the foundations towards job stability. The competitiveness of such a research environment will not promote more expensive, longer-term projects that may be narrower in terms of focus yet have the sufficient depth to provide more rigorous and reproducible findings. To achieve truly informative and reliable science, a balance has to be struck between *sufficiency, thoroughness and value for money* which is not necessarily something that can be rushed.

Science is seen as ‘self-correcting’ due to the premise that it is built on the replication of earlier work [4]. Yet for this function to occur there is a requirement to validate key data, prior to its use as a foundation to advance the research question. However, there is limited incentive from journals and funding bodies to pursue such studies, or to publish results that may contradict or confirm previous papers [3]. This is often a vital service to avoid wasting money and effort, and provide confidence in future scientific output. However, some top-tier journals are beginning to recognise the importance of this aspect of research by providing journal space for this type of study, particularly where they confirm or refute important and/or controversial findings [3]. The usefulness of negative findings or contradictory data should also be adopted more widely in peer-reviewed journals [10]. We acknowledge that *Particle and Fibre Toxicology* also uses novelty often as a key selection criterion, as it should do. However, reviewers are encouraged to give investigators a fair chance when submitting replication studies, if clearly warranted and properly substantiated.

## The solution?

How do we address this apparent conflict? Collins and Tabak of the National Institutes of Health (NIH) point to community responsibility and propose a range of approaches to tackle the various levels in the hierarchy of scientific research that may have contributed to the increasing concern in this area [4]. Their advice ranges from journals being encouraged to devote more space to research conducted in an exemplary manner, as well as those that report negative findings [10]. They also highlight that *Nature Publishing Group* have abolished restrictions on the length of Methods sections to ensure the reporting of essential experimental detail [3]. They also suggest a move away from using “arbitrary surrogates” of an investigator’s scientific contribution and future potential when considering job promotion or grant awards. In particular they emphasise that the use of relatively uninformative measures such as the numbers of grants awarded or numbers of publications in journals with high impact factors may underestimate the true value of a researcher’s output that could be low in number, but high in impact in a broader sense. Interestingly the NIH is also considering strategies that provide greater stability for investigators (at crucial career stages) by offering more flexibility and longer project durations within grant mechanisms to reduce the (perceived?) pressure to generate positive results and numerous papers within short periods of time. This is an interesting approach but also one that would need further support from academic departments through a greater focus on career development and a broader means of evaluating what an individual brings to their department.

Whilst the approaches put forward by Collins and Tabak are largely from a top-down perspective, the editorial by Gary Miller [1] discusses a more fundamental, ground-up approach for improving the rigour of day-to-day scientific research. He suggests further encouragement of researcher awareness of unintended bias by, as Miller puts it, evoking the spirit of Karl Popper and attempting to disprove what we believe to be true [1]. In particular, to evoke a mind-set that reduces confirmation bias where an experimenter (purposely, or inadvertently) directs the course of study to confirm what they believe to be true. Miller suggests that we too often attempt to rid our experiments of any sort of experimental variability through good intention, but this in itself limits reproducibility. Should we instead ‘add in a little variability’, i.e. ensure reproducibility of findings are maintained despite variables such as differences between lots of reagents, different experimenters and laboratories performing the test, etc? However the presence of variability is already well entrenched in biological sciences where the complexity, instability and unpredictability of biological systems is arguably more profound than within other, more fundamental disciplines such as chemistry and physics [5]. This is encapsulated within ‘Harvard’s law’ (and perhaps even taped-up as a notice in your own cell culture laboratories) which states that “Under the most rigorously controlled conditions of pressure, temperature, volume, humidity, and other variables the organism will do as it damn well pleases”. The field of particle and fibre toxicology, and especially nanotoxicology, adds an additional layer of complexity through the addition of surprisingly dynamic physicochemical properties of particles, dispersion states, etc. once such particles are employed in the biological model of interest. Many particle/nano- journals now require very high standards of independent characterisation of the test compounds and reporting of methods, properties and sources of materials as standard; not just a reliance of citation of other publications with similar methodology. In a sense, one should characterize materials in such a way that the hypothesis of the study can be addressed and that peers will be able to reproduce the exposures.

The crux of this is the use of replication and rigorous confirmation of an observed endpoint and this takes time, funds and, in the case of *in vivo* studies, may also be considered as non-ethical. Stakeholders (e.g. funding bodies) have a responsibility to ensure that high-output strategies do not represent a false economy due to a lack of reproducibility. However the onus clearly lies on the researchers also; what is required to prove, with confidence, your results? For example, does significance at n = 3–4 truly provide conclusive proof of a hypothesis? Recently Johnson [1],[11] suggested that the current standard of p < 0.05 for statistical significance is a major source of irreproducibility in both big and small data sets alike. Aiming for a more stringent threshold level of significance (such as 0.01 or 0.005) and greater use of dose-response curves rather than single dose studies would undoubtedly require more replicates. However, it would also push researchers not to simply do ‘just enough’ to demonstrate a positive finding, but to ensure that the biological significance of findings are better represented across the spectrum of biological variability. At very least, our conclusions and the adoption of bold statements should be reflective of the statistical evidence; where results are barely significant, it may be more prudent to suggest that the results are indicative of something rather than that they confidently state fact.

Understandably, many scientists will be less inclined to risk undermining their prospects of grant funding and career progression by increasing their proposal costs and reducing publication rates. However, there are bottom-up approaches that can be taken. These range from a more conscientious approach to experimental design, analysis and reporting (including training junior staff to be similarly dedicated) to testing our hypotheses in scientific forums, be that through on-line media or by formal peer review. This editorial does not propose to offer all of the solutions but simply add voice to the growing concern and call for a collective response to the issue and stimulate debate on the topic in all forums because as stated by Gary Miller “Toxicology just is not toxicology without reproducibility” [1] and we whole heartedly agree. There is no panacea but allow us to offer five practical points to consider during study design through to reporting the findings, from the perspective of particle toxicology.

1. **Be specific.** For example, what is your sample? If it’s graphene, then is it actually graphene i.e. a monolayer, or is it few layer graphene, or graphite platelets? If they are ambient particles, where and how were they collected (date, time, weather conditions etc.)? State this, it is important - later studies may not replicate your results simply due to inadvertently testing the non-similar materials i.e. comparing apples and oranges.

2. **Characterise, characterise, characterise.** Only with independent characterisation do you truly know what you are testing and allow others to reproduce your findings. Particle characteristics will undoubtedly be a key driver of bioactivity. Characterise in relation to your hypothesis or research question - do not characterise because there is a default list of parameters that you need to check.

3. **Use statistics to question your results rather than simply confirming a theory.** Ask yourself, is barely statistically significant really biologically relevant? Can you support/validate an entire hypothesis or more controversially, a provocative statement using P < 0.05?

4. **Write your hypothesis in advance of running the experiment.** It should be clear from the experimental design how you have tried to disprove this. If your theory is robust it should survive your best efforts to challenge it.

5. **Take a ‘belts and braces’ approach to confirming findings.** Use multiple approaches, doses, time points, replicates and controls. You don’t want to be caught with your trousers down.

## Competing interests

The authors declare that they have no competing interests.

## References

[B1] Miller GW Improving reproducibility in toxicology Toxicol Sci 2014 139 1 3 10.1093/toxsci/kfu050 24747876

[B2] Must try harder Nature 2012 483 509 10.1038/483509a 22460859

[B3] **Announcement: reducing our irreproducibility.** *Nature* 2013, **496:**398.

[B4] Collins FS Tabak LA Policy: NIH plans to enhance reproducibility Nature 2014 505 612 613 10.1038/505612a 24482835 PMC4058759

[B5] Gori GB Elusive reproducibility Regul Toxicol Pharmacol 2014 69 279 280 10.1016/j.yrtph.2014.05.020 24882687

[B6] Cumming G Fidler F Vaux DL Error bars in experimental biology J Cell Biol 2007 177 7 11 10.1083/jcb.200611141 17420288 PMC2064100

[B7] Pusztai L Hatzis C Andre F Reproducibility of research and preclinical validation: problems and solutions Nat Rev Clin Oncol 2013 10 720 724 24080600 10.1038/nrclinonc.2013.171

[B8] Vaux DL Research methods: know when your numbers are significant Nature 2012 492 180 181 23235861 10.1038/492180a

[B9] Laurent S Burtea C Thirifays C Hafeli UO Mahmoudi M Crucial ignored parameters on nanotoxicology: the importance of toxicity assay modifications and “cell vision” PLoS One 2012 7 e29997 10.1371/journal.pone.0029997 22253854 PMC3254623

[B10] Hankin S Boraschi D Duschl A Lehr CM Lichtenbeld H Towards nanotechnology regulation–publish the unpublishable Nano Today 2011 6 228 231 10.1016/j.nantod.2011.03.002

[B11] Johnson VE Revised standards for statistical evidence Proc Natl Acad Sci U S A 2013 110 19313 19317 10.1073/pnas.1313476110 24218581 PMC3845140

